# The impact of PM_2.5_ component exposure during early pregnancy on congenital heart disease: evidence from Guangdong, China

**DOI:** 10.1038/s41598-026-59940-7

**Published:** 2026-07-28

**Authors:** Yanbing Chen, Peiming Su, Qixian Gu, Huili Wei, Jialing Qiu, Guangming Tan

**Affiliations:** 1 Medical Genetic Center, Guangdong Women and Children Hospital, 521 Xingnan Road, Panyu District, Guangzhou, 511442 China; 2 School of Public Health, Guangzhou Medical University, 1 Xinzao Road, Xinzao Town, Panyu District, Guangzhou, 511436 China; 3 Department of Medical Information and Statistics, Guangdong Women and Children Hospital, 521 Xingnan Road, Panyu District, Guangzhou, 511442 China; 4 Department of Public Health and Preventive Medicine, School of Medicine, Jinan University, Guangzhou, 510632 China; 5 Department of Human Resources, Guangdong Women and Children Hospital, 521 Xingnan Road, Panyu District, Guangzhou, 511442 China

**Keywords:** Cardiology, Diseases, Environmental sciences, Medical research, Risk factors

## Abstract

Limited is known about how the components of particulate matter with aerodynamic diameter < 2.5 μm (PM_2.5_) are related to congenital heart disease (CHD). We aimed to demonstrate the relationship between exposure to PM_2.5_ components during early pregnancy and the risk of CHD. This study included 2,601,300 live births and stillbirths in Guangdong, China from 2014 to 2017. Satellite remote sensing data of PM_2.5_ components were collected, including organic matter (OM), sulfate (${\mathrm{S}\mathrm{O}}_{4}^{2-}$), nitrate (${\mathrm{N}\mathrm{O}}_{3}^{-}$), ammonium (${\mathrm{N}\mathrm{H}}_{4}^{+}$), and black carbon (BC). Unconditional logistic regression models were constructed to estimate the relationship of each PM_2.5_ component to CHD. The quantile g-computation method was used to evaluate the contribution weight of each PM_2.5_ component in relation to CHD in the exposure mixture. The estimated effect size of each PM_2.5_ component peaked in the first week of pregnancy. Among them, ${\mathrm{S}\mathrm{O}}_{4}^{2-}$ exhibited the strongest estimated effect (OR: 2.23, 95% CI 2.19–2.27), followed by BC (OR: 1.95, 95% CI 1.91–1.98). The joint exposure analysis revealed that the estimated weights of ${\mathrm{S}\mathrm{O}}_{4}^{2-}$ and BC were 86.78% and 13.22%, respectively. These findings suggest that exposure to PM_2.5_ components during early pregnancy increases the risk of CHD in the offspring, and ${\mathrm{S}\mathrm{O}}_{4}^{2-}$ may be the primary PM_2.5_ component responsible for this elevated risk.

**Supplementary Information:**

The online version contains supplementary material available at https://doi.org/10.1038/s41598-026-59940-7.

## Introduction

Congenital heart disease (CHD) is a group of birth defects characterized by abnormal heart and great vessel development during embryogenesis, and one of the most common birth defects^1^. If severe CHD is not detected and treated promptly after birth, a large proportion of affected infants die from severe complications within the first year of life, significantly increasing mortality rate^2^. Common CHD subtypes include ventricular septal defect, atrial septal defect, patent ductus arteriosus, and tetralogy of Fallot^3^. From 1990 to 2021, neonatal CHD prevalence rose gradually, with a global average of 2.10 per 1000 live births^4^. Notably, the prevalence is higher in China, reaching 8.44 per 1000 live births^5^. Globally, CHD causes up to 217,000 deaths annually, with a one-year survival rate of only 85.8% for severe cases, posing a major threat to newborn health^6,7^. The etiology of CHD is complex and incompletely understood, and multiple studies show that it arises from genetic-environmental interplay. Genetic syndromes (e.g., gene mutations and chromosomal abnormalities) account for approximately 40% of CHD cases, while over 60% relate to environmental factors, indicating their key role in CHD occurrence^8,9^.

Recent epidemiological studies have confirmed that prenatal severe particulate matter with aerodynamic diameter < 2.5 μm (PM_2.5_) exposure is significantly associated with increased CHD risk in offspring. However, existing research exhibits certain heterogeneity in terms of effect size, CHD subtypes, and key exposure time windows^10,11^. Mechanism studies indicate that exposure to PM_2.5_ may be involved in the occurrence of adverse pregnancy outcomes by inducing placental inflammatory responses, with elevated levels of inflammatory factors such as IL-6 and TNF-α observed in chorionic villi tissue^12^. In addition, animal experiments have shown that related oxidative stress pathways and endoplasmic reticulum stress may participate in the cardiac developmental toxicity caused by PM_2.5_^13,14^. Therefore, PM_2.5_ may be one of the important environmental risk factors for CHD, but its key toxic components and critical exposure window still need further research and clarification.

PM_2.5_ has a complex chemical composition, including organic matter (OM), sulfate (${\mathrm{S}\mathrm{O}}_{4}^{2-}$), nitrate (${\mathrm{N}\mathrm{O}}_{3}^{-}$), ammonium (${\mathrm{N}\mathrm{H}}_{4}^{+}$), and black carbon (BC)^15^. Its large surface area allows it to act as a carrier for various pollutants, enhancing its compositional diversity and health risks. Currently, there are only two studies on PM_2.5_ components and CHD, making it difficult to draw systematic conclusions^16,17^. Both studies link elevated maternal PM_2.5_ exposure (periconceptional, pre-pregnancy, early pregnancy) to higher offspring CHD risk in China, but differ in many aspects (component selection, exposure windows, CHD subtypes, and effect modes). For example, a large national study identified OM as the dominant contributor during 12 months pre-pregnancy, 6 months pre-pregnancy, and early pregnancy, while a Chongqing study highlighted synergistic effects of BC and UM during 3 months pre-pregnancy and early pregnancy. Therefore, investigating the impact of distinct PM_2.5_ components during pregnancy on the CHD risk and critical exposure windows is essential to clarify mechanisms and guide targeted interventions/policies.

With China’s rapid economic development, PM_2.5_ pollution has occurred frequently over the past decade, threatening public health^18^. Since 2013, the government has strengthened PM_2.5_ control, leading to significant improvements in air quality^19^. Nevertheless, PM_2.5_ remains an important health hazard due to its small particle size and strong penetrating power, especially its potential and lasting risks to the respiratory and cardiovascular system^20^. Guangdong, located at the south of China, had 1.133 million livebirths in 2024, providing a large sample size for studying adverse pregnancy outcomes^21^. As a typical Chinese region with advanced economy, dense population, and rapid urbanization, Guangdong has complex PM_2.5_ pollution from industrial emissions, vehicle exhaust, biomass burning, regional transport, and secondary formation^22^. Its PM_2.5_ composition is complex with significant spatiotemporal heterogeneity, providing a suitable exposure gradient to identify key toxic components and their independent/joint effects. Additionally, Guangdong’s robust maternal-child health and atmospheric monitoring networks offer high-quality data for exposure assessment and CHD case identification^23^. Therefore, we conducted a retrospective analysis of neonatal birth data in Guangdong (2014–2017) to examine the association between major PM_2.5_ components and CHD, and identify key toxic components. The findings will help promote optimal birth outcomes and reduce CHD burden on families and society.

## Methods

### Study population

The data were from the Guangdong provincial birth registration database, in which birth information was obtained from all midwifery clinics and hospitals in Guangdong Province. The dataset includes birth date, maternal age, mode of delivery, birth weight, fetal outcome, birth defects, neonatal gender, gestational age, gravidity, parity and mother’s residential district/county.

**Fig. 1:**
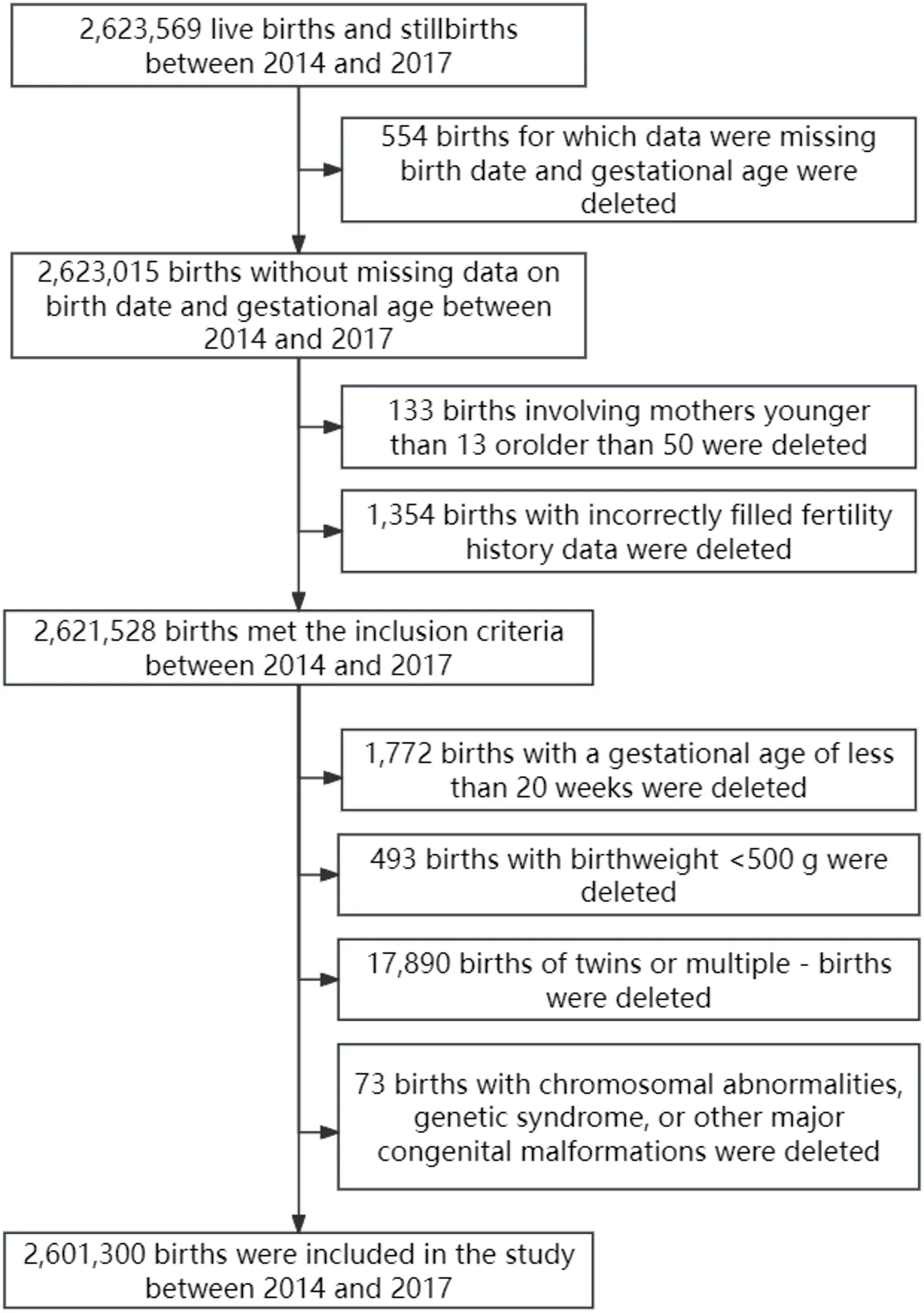
The flow chart of the study population screening.

We collected 2,623,569 vaginal live births and stillbirths in 21 cities in Guangdong Province from January 1, 2014 to December 31, 2017. Furthermore, the following birth data were excluded: (1) 554 cases where neonatal birth date or gestational age data were missing; (2) 133 mothers aged > 50 years old or <13 years old; (3) 1354 cases with incorrectly filled maternal pregnancy history data; (4) 1772 cases of gestational age less than 20 weeks; (5) 493 cases of birth weight < 500g; (6) 17,890 cases of twins or multiple births ; (7) 73 cases with chromosomal abnormalities, other congenital malformations, or maternal teratogenic diseases. As a result, a total of 2,601,300 newborns were finally included for analysis. Figure 1 showed the flow chart of the study population selection.

### The PM_2.5_ components exposure data

The daily average concentration data of PM_2.5_ components (OM, ${\mathrm{S}\mathrm{O}}_{4}^{2-}$, ${\mathrm{N}\mathrm{O}}_{3}^{-}$, ${\mathrm{N}\mathrm{H}}_{4}^{+}$, BC) were obtained from the near real-time tracking dataset of atmospheric components in China (http://tapdata.org.cn). Based on multi-source data sets and machine learning algorithms, the TAP database integrates satellite remote sensing data, meteorological field, chemical transport model simulation, ground measurement and land use information^24,25^. Compared with other data sources, it has high accuracy, and it provides a multi-scale, complete spatial and temporal coverage, and near real-time gaseous pollutant concentration data set in China. The PM_2.5_ component concentrations estimated by the TAP database with a spatial resolution of 10 km closely approximates ground-based measurements. The correlation coefficients of daily average concentrations from 2013 to 2020 are 0.67 ~ 0.80, and most of the standard deviations are within ± 20%. We geographically encoded the residence area during the pregnancy of each participant’s mother to the district/county level, and calculated the mother’s last menstrual date based on their birth date and gestational age. Then the daily average PM_2.5_ measurements in the TAP database were linked to each mother based on the geographical coding and pregnancy date. Existing evidence demonstrates that early pregnancy (especially 3–8 weeks of gestation) is a highly sensitive window period for human cardiovascular system development, during which the embryo is susceptible to teratogenic factors, environmental interference and organ morphology abnormalities^26^. Accumulative epidemiological studies have suggested that PM_2.5_ exposure in early pregnancy may be associated with an increased risk of CHD in offspring, with 3–10 weeks after conception confirmed to be validated as the critical exposure time window^27–29^. Given that these critical time windows all fall within early gestation, we therefore defined the first trimester (0–12 gestational weeks) as the susceptible window in this study.

### Covariates

The covariates were maternal age, gravidity, parity, conception season, gestational age, neonatal gender, and temperature. The related medical information was available in Guangdong provincial birth registration database, and were recorded by the medical staff. For further analysis, maternal age was reclassified into <20,20~24,25~29,30~34, and ≥35; gravidity was reclassified into multigravida and primigravida; parity was reclassified into multipara and primipara; conception date was reclassified into spring, summer, autumn and winter. The temperature data were obtained from the daily temperature data set of China from the National Tibetan Plateau Scientific Data Center. For the daily average temperature, the RMSE range was 0.35 °C~1.00 °C, the MAE range was 0.27 °C ~0.68 °C, and the R_2_ range was 0.99~1.00. We geographically encoded the residence area during the pregnancy of each participant’s mother to the district/county level, and calculated the mother’s last menstrual date based on their birth date and gestational age. Then the daily average temperature measurements in the database were linked to each mother based on the geographical coding and pregnancy date.

### The diagnosis of CHD

The outcome of this study was CHD. It was diagnosed by transthoracic color Doppler echocardiography, catheter insertion, or surgery, combined with clinical symptoms of the case. The diagnosis was carried out by two or more associate chief physicians with experience in fetal echocardiography examination in the hospital with prenatal screening and diagnosis qualifications. All cases of CHD were classified according to the International Statistical Classification of Diseases and Related Health Problems, Tenth Edition (ICD-10, Q20-Q28). The subtypes included ventricular septal defect (VSD, Q21.0), atrial septal defect (ASD, Q21.1), patent ductus arteriosus (PDA, Q25.0), Tetralogy of Fallot (ToF, Q21.3), pulmonary valve stenosis (vPS, Q22.1), atrioventricular septal defect (AVSD, Q21.2), right ventricular double outlet (DORV, Q20.1), or other cardiac structural abnormalities.

### Statistical analysis

We first described the demographic characteristics between infants with and without CHD, respectively. The differences between groups were compared using chi-square test or t test . We also described the distribution level of maternal exposure to the pollutants during the study period. The correlation between PM_2.5_ components was assessed using the Spearman rank correlation test. We used an unconditional multivariate logistic regression model to examine the association between the maternal air pollutants exposure and CHD risk in offspring. The exposure concentration of PM_2.5_ components was used as an independent variable, and the CHD was used as a dependent variable. The following important covariates were adjusted, including maternal age, gravidity, parity, conception season, gestational age, neonatal gender, and temperature. For each PM_2.5_ component, we estimated the corresponding odds ratios (ORs) and 95% confidence intervals (CIs) during different pregnancies for each increase in the interquartile range (IQR) of air pollutant concentration. In addition, the residential region of each woman was categorized into Eastern Guangdong, Northern Guangdong, Western Guangdong and Pear River Delta. A stratified analysis was conducted based on the residential regions of pregnant women. In view of the high correlation between the components of PM_2.5_, we used the quantile g-computation (QGC) method to examine the joint effect of a PM_2.5_ mixture on CHD. The weight of each major PM_2.5_ component was also calculated to understand its proportional contribution to the overall effect estimate of exposure mixture^30^. Two sensitivity analyses were carried out to test the robustness of the results in the main analyses. First, the stillbirth infants were excluded in the fully adjusted model; second, we constructed the weighted quantile sum (WQS) regression model to assess the weights and the overall mixture effect of PM_2.5_ exposure on CHD in infants. We used R4.4.1 for statistical analysis, with a test level *α* set at 0.05 on both sides.

## Results

### Demographic characteristics of participants

A total of 2,601,300 newborns or fetuses (including 2,596,979 live births and 4321 stillbirths) were included in this study, of which 17,812 had CHD (Table 1). A majority of mothers were under 35 years old (92.90%), and 55.80% of the infants with CHD were male. Compared to the mothers of the infants without CHD, those of the infants with CHD were more likely to be over 35 years old (15.02% vs. 7.05%) and not conceive in winter (23.63% vs. 29.04%). More than half of the pregnant women had experienced previous pregnancies (58.32%) and deliveries (52.75%).

**Table 1:** Demographic characteristics of participants.

Characteristics		Total (n = 2,601,300)	Without CHD (n = 2,583,488)	CHD (n = 17,812)	*P*
Gestational age (weeks, mean)		38.94 (1.49)	39.0 (1.40)	36.7 (4.40)	< 0.001
Maternal age	< 20	142,291 (5.47)	141,894 (5.49)	397 (2.23)	< 0.001
	20–24	761,926 (29.29)	758,846 (29.37)	3080 (17.29)	
	25–29	1,039,466 (39.96)	1,032,401 (39.96)	7065 (39.66)	
	30–34	472,798 (18.18)	468,203 (18.12)	4595 (25.80)	
	≥ 35	184,819 (7.10)	182,144 (7.05)	2675 (15.02)	
Gravidity, n (%)	Multigravida	1,516,954 (58.32)	1,506,168 (58.30)	10,786 (60.55)	< 0.001
	Primigravida	859,690 (33.05)	853,101 (33.02)	6589 (36.99)	
	Missing	224,656 (8.64)	224,219 (8.68)	437 (2.45)	
Parity, n (%)	Multipara	1,372,064 (52.75)	1,365,143 (52.84)	6921 (38.86)	< 0.001
	Primipara	1,012,289 (38.91)	1,001,879 (38.78)	10,410 (58.44)	
	Missing	216,947 (8.34)	216,466 (8.38)	481 (2.70)	
Gender, n (%)	Male	1,361,216 (52.33)	1,351,277 (52.30)	9939 (55.80)	< 0.001
	Female	1,239,269 (47.64)	1,231,443 (47.67)	7826 (43.94)	
	Unknown or missing	815 (0.03)	768 (0.03)	47 (0.26)	
Conception season, n (%)	Spring (March–May)	630,177 (24.23)	625,678 (24.22)	4499 (25.26)	< 0.001
	Summer (June–August)	576,929 (22.18)	572,425 (22.16)	4504 (25.29)	
	Autumn (September–November)	639,848 (24.60)	635,248 (24.59)	4600 (25.83)	
	Winter (December–February)	754,346 (29.00)	750,137 (29.04)	4209 (23.63)	

### Distribution and correlation analysis of air pollutants

In the first three months of pregnancy, the concentration distribution of PM_2.5_ components is presented in Table 2, with significant variations observed in the exposure levels of different chemical components. In terms of the total mass proportion of PM_2.5_, OM accounted for the largest share, followed by ${\mathrm{S}\mathrm{O}}_{4}^{2-}$, ${\mathrm{N}\mathrm{O}}_{3}^{-}$, ${\mathrm{N}\mathrm{H}}_{4}^{+}$ and BC. Throughout the study, their mean (standard deviation) of concentrations were 8.04 (3.58), 6.05 (2.54), 4.76 (3.11), 3.76 (2.26) and 1.74 (0.75) μg/m^3^, respectively. In addition, the study found a significant pairwise correlation among the components of PM_2.5_ (the correlation coefficient r = 0.681–0.989), where ${\mathrm{N}\mathrm{O}}_{3}^{-}$ and ${\mathrm{N}\mathrm{H}}_{4}^{+}$ had the strongest correlation (r = 0.989), as shown in Table 3.

**Table 2:** Maternal exposure levels of PM_2.5_ components and ambient temperature.

Pollutant	Mean	SD	Minimum	Maximum	P25	P50	P75	IQR
OM (μg/m^3^)	8.04	3.58	0.73	34.82	5.45	7.31	9.81	4.36
${\mathrm{S}\mathrm{O}}_{4}^{2-}$(μg/m^3^)	6.05	2.54	0.69	26.36	4.16	5.62	7.45	3.29
${\mathrm{N}\mathrm{O}}_{3}^{-}$(μg/m^3^)	4.76	3.11	0.45	28.32	2.53	3.83	6.14	3.61
${\mathrm{N}\mathrm{H}}_{4}^{+}$(μg/m^3^)	3.76	2.26	0.40	21.60	2.08	3.22	4.91	2.83
BC (μg/m^3^)	1.74	0.75	0.13	8.48	1.21	1.60	2.12	0.91
Temperature (°C)	22.09	5.78	0.52	32.34	17.23	23.29	27.26	10.03

*OM, organic matter; ${\mathrm{S}\mathrm{O}}_{4}^{2-}$, sulfate; ${\mathrm{N}\mathrm{O}}_{3}^{-}$, nitrate; ${\mathrm{N}\mathrm{H}}_{4}^{+}$, ammonium; BC, black carbon; SD, standard deviation; IQR, interquartile range.

**Table 3:** Correlation between PM_2.5_ components.

Air pollutants	OM	${\mathrm{S}\mathrm{O}}_{4}^{2-}$	${\mathrm{N}\mathrm{O}}_{3}^{-}$	${\mathrm{N}\mathrm{H}}_{4}^{+}$	BC
OM	1	0.946*	0.799*	0.775*	0.938*
${\mathrm{S}\mathrm{O}}_{4}^{2-}$		1	0.838*	0.834*	0.940*
${\mathrm{N}\mathrm{O}}_{3}^{-}$			1	0.989*	0.681*
${\mathrm{N}\mathrm{H}}_{4}^{+}$				1	0.685*
BC					1

*OM, organic matter; ${\mathrm{S}\mathrm{O}}_{4}^{2-}$, sulfate; ${\mathrm{N}\mathrm{O}}_{3}^{-}$, nitrate; ${\mathrm{N}\mathrm{H}}_{4}^{+}$, ammonium; BC, black carbon. *: *P* < 0.05.

### The association between PM_2.5_ components and CHD

Table 4 shows the ORs and 95% CIs of weekly exposure to PM_2.5_ components associated with CHD risk. Specifically, all five major pollutants of PM_2.5_ were associated with the highest risk of CHD in the first week of pregnancy. Among them, ${\mathrm{S}\mathrm{O}}_{4}^{2-}$ had the largest estimated effect (OR = 2.23, 95% CI 2.19–2.27), followed by BC (OR = 1.95, 95% CI 1.91–1.98), OM (OR = 1.89, 95% CI 1.86–1.93), ${\mathrm{N}\mathrm{O}}_{3}^{-}$ (OR = 1.57, 95% CI 1.54–1.61) and ${\mathrm{N}\mathrm{H}}_{4}^{+}$ (OR = 1.44, 95% CI 1.40–1.48).

**Table 4:** Logistic regression analysis of PM_2.5_ components and congenital heart disease in different pregnancy periods.

Gestational weeks	OM	${\mathrm{S}\mathrm{O}}_{4}^{2-}$	${\mathrm{N}\mathrm{O}}_{3}^{-}$	${\mathrm{N}\mathrm{H}}_{4}^{+}$	BC
	OR (95% CI)	OR (95% CI)	OR (95% CI)	OR (95% CI)	OR (95% CI)
1	1.89 (1.86–1.93)	2.23 (2.19–2.27)	1.57 (1.54–1.61)	1.44 (1.40–1.48)	1.95 (1.91–1.98)
2	1.87 (1.83–1.90)	2.22 (2.18–2.26)	1.55 (1.51–1.58)	1.43 (1.39–1.46)	1.94 (1.91–1.97)
3	1.83 (1.80–1.87)	2.19 (2.15–2.23)	1.51 (1.48–1.55)	1.40 (1.37–1.44)	1.92 (1.89–1.95)
4	1.78 (1.75–1.81)	2.13 (2.10–2.17)	1.47 (1.43–1.50)	1.36 (1.32–1.39)	1.87 (1.84–1.90)
5	1.72 (1.69–1.75)	2.05 (2.01–2.08)	1.41 (1.38–1.45)	1.30 (1.27–1.33)	1.81 (1.78–1.84)
6	1.73 (1.70–1.75)	2.05 (2.02–2.09)	1.43 (1.40–1.47)	1.30 (1.27–1.34)	1.81 (1.78–1.84)
7	1.70 (1.67–1.73)	2.01 (1.97–2.04)	1.41 (1.38–1.44)	1.29 (1.26–1.32)	1.77 (1.75–1.80)
8	1.71 (1.68–1.74)	2.00 (1.97–2.04)	1.41 (1.38–1.44)	1.30 (1.27–1.33)	1.79 (1.76–1.82)
9	1.70 (1.67–1.72)	2.00 (1.96–2.03)	1.37 (1.34–1.40)	1.26 (1.23–1.29)	1.79 (1.76–1.82)
10	1.66 (1.63–1.69)	1.97 (1.94–2.01)	1.32 (1.29–1.35)	1.21 (1.18–1.24)	1.77 (1.75–1.80)
11	1.69 (1.66–1.72)	2.03 (1.99–2.07)	1.35 (1.32–1.38)	1.25 (1.22–1.28)	1.80 (1.77–1.83)
12	1.73 (1.70–1.76)	2.10 (2.06–2.14)	1.36 (1.33–1.39)	1.25 (1.22–1.28)	1.86 (1.83–1.89)

*OM, organic matter;${\mathrm{S}\mathrm{O}}_{4}^{2-}$, sulfate;${\mathrm{N}\mathrm{O}}_{3}^{-}$, nitrate;${\mathrm{N}\mathrm{H}}_{4}^{+}$, ammonium; BC, black carbon. All the above models were adjusted according to the age of the mother, gravidity, parity, neonatal gender, gestational age, conception season and temperature.

**Fig. 2:**
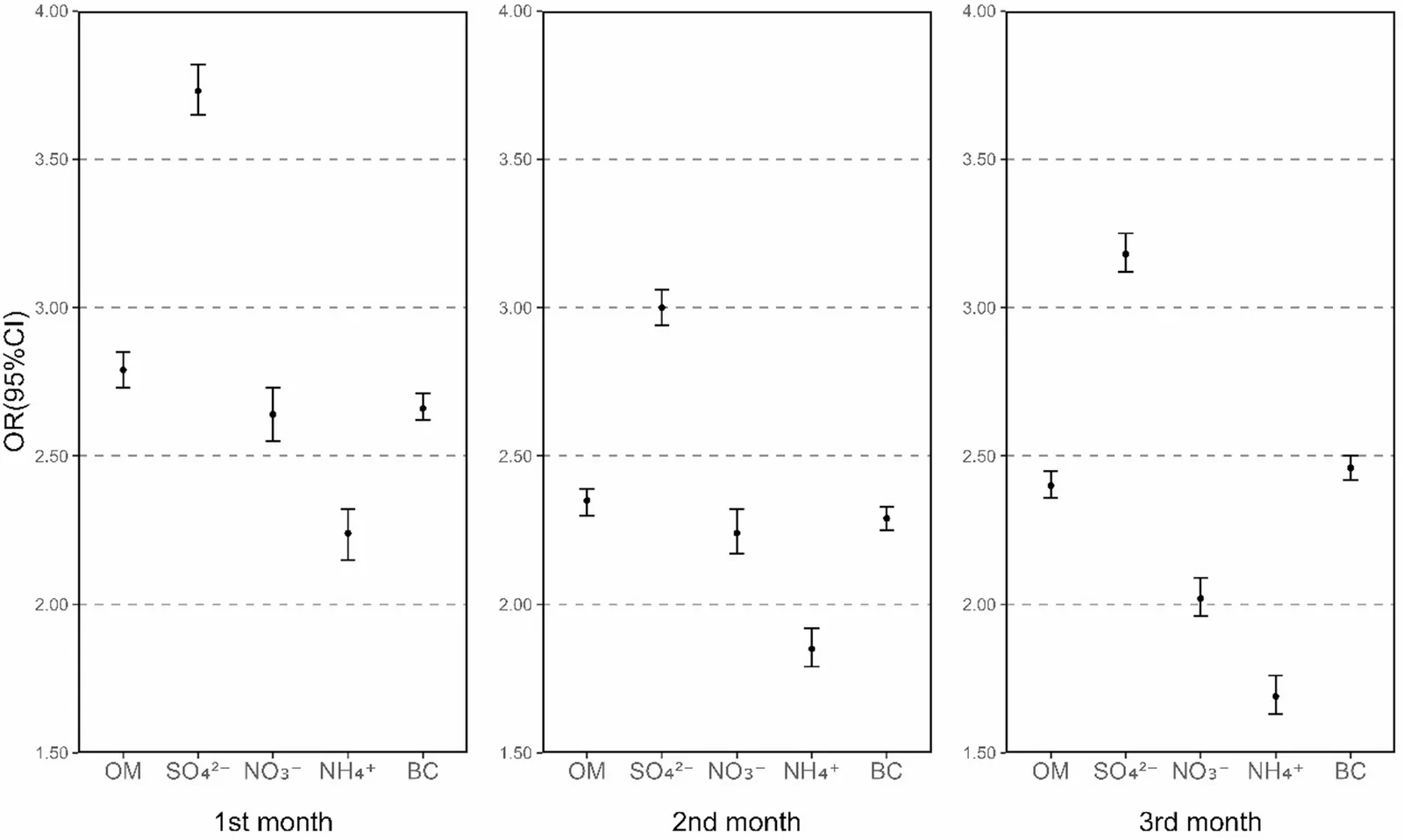
The odds ratio and 95% confidence interval of congenital heart disease for each increase in interquartile range of PM_2.5_ concentration during the first three months of pregnancy. *Note* OM, organic matter; ${\mathrm{S}\mathrm{O}}_{4}^{2-}$, sulfate; ${\mathrm{N}\mathrm{O}}_{3}^{-}$, nitrate; ${\mathrm{N}\mathrm{H}}_{4}^{+}$, ammonium; BC, black carbon. All the above models were adjusted according to the age of the mother, gravidity, parity, neonatal gender, gestational age, conception season and temperature.

Figure 2 shows the ORs and 95% CIs of monthly PM_2.5_ exposure associated with CHD risk. The ORs of the three pollutants OM, ${\mathrm{S}\mathrm{O}}_{4}^{2-}$, and BC were the highest in the first month, decreased in the second, and rose again in the third, exhibiting a consistent pattern when analyzed on a weekly basis. Unlike other pollutants, the risk of CHD under the exposure of two pollutants ${\mathrm{N}\mathrm{O}}_{3}^{-}$ and ${\mathrm{N}\mathrm{H}}_{4}^{+}$ showed a decreasing trend with an increasing month of pregnancy.

### The contribution weight of PM_2.5_ components

**Fig. 3:**
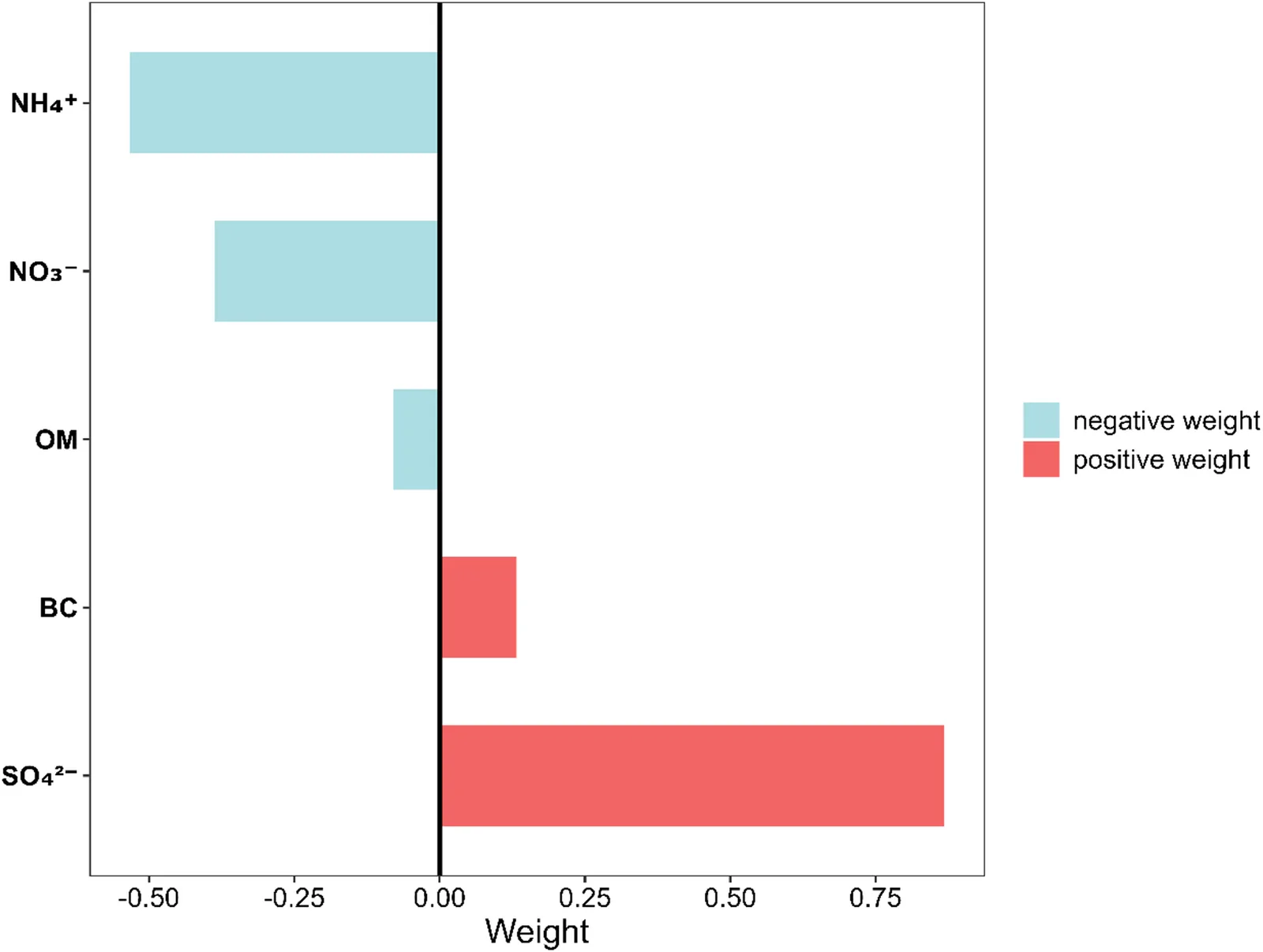
The contribution weights of the five main components of PM_2.5_ to the overall effect on congenital heart disease.

In the joint exposure analysis, the estimated weights of ${\mathrm{S}\mathrm{O}}_{4}^{2-}$ and BC for CHD were positive, of which ${\mathrm{S}\mathrm{O}}_{4}^{2-}$ accounted for 86.78% and BC accounted for 13.22% (Fig. 3). However, the estimated weights of OM, ${\mathrm{N}\mathrm{O}}_{3}^{-}$ and ${\mathrm{N}\mathrm{H}}_{4}^{+}$ for CHD were negative, indicating that these three components have an offsetting effect on the total effect in mixed exposure. Since the effect of PM_2.5_ exposure on CHD was positive, this study focused on ${\mathrm{S}\mathrm{O}}_{4}^{2-}$ and BC with positive weights. ${\mathrm{S}\mathrm{O}}_{4}^{2-}$ had the highest weight among the five main components of PM_2.5_, indicating that ${\mathrm{S}\mathrm{O}}_{4}^{2-}$ is the most important component.

### Subgroup analyses

**Fig. 4:**
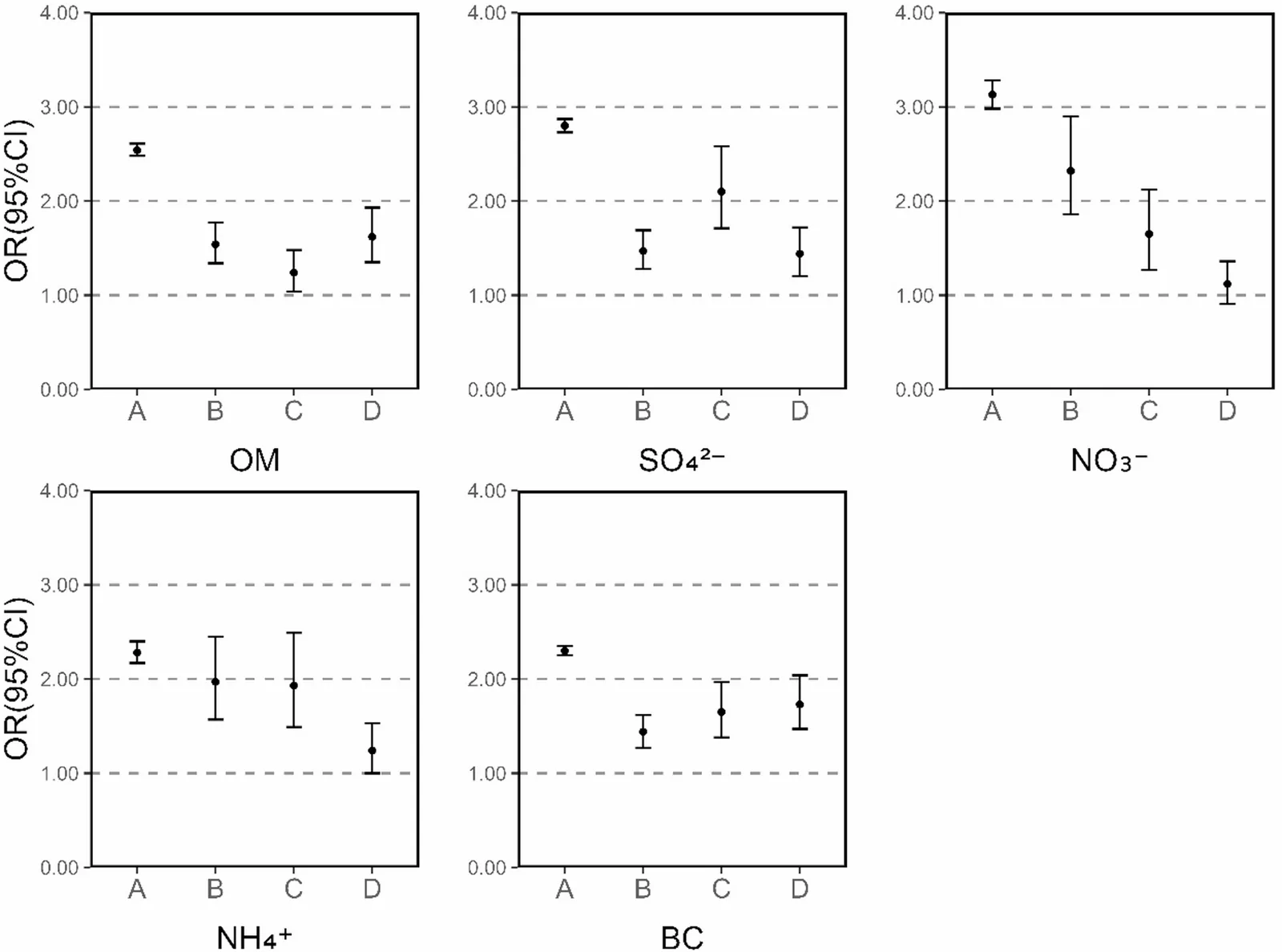
The odds ratio and 95% confidence interval of congenital heart disease with increased interquartile range in PM_2.5_ concentration in different regions. *Note* A, Pearl River Delta; B, Eastern Guangdong; C, Western Guangdong; D, Northern Guangdong. OM, organic matter; ${\mathrm{S}\mathrm{O}}_{4}^{2-}$, sulfate; ${\mathrm{N}\mathrm{O}}_{3}^{-}$, nitrate; ${\mathrm{N}\mathrm{H}}_{4}^{+}$, ammonium; BC, black carbon. The model was adjusted according to maternal age, gravidity, parity, neonatal gender, gestational age, conception season and temperature.

Figure 4 shows the association between PM_2.5_ components and CHD stratified by pregnant women’s residential area. We found that the association between PM_2.5_ components and CHD differed across regions of Guangdong. Specifically, pregnant women in the Pearl River Delta region have the highest susceptibility to PM_2.5_. Moreover, pregnant women in western Guangdong showed the lowest susceptibility to OM (OR = 1.24, 95% CI 1.04–1.48) and the second highest susceptibility to ${\mathrm{S}\mathrm{O}}_{4}^{2-}$(OR = 2.10, 95% CI 1.71–2.58), whereas the opposite was true for those in northern Guangdong (OM : OR = 1.62, 95% CI 1.35–1.93; ${\mathrm{S}\mathrm{O}}_{4}^{2-}$: OR = 1.44, 95% CI 1.20–1.72). Meanwhile, pregnant women in eastern Guangdong were more susceptible to ${\mathrm{N}\mathrm{O}}_{3}^{-}$ (OR = 2.32, 95% CI 1.86–2.90) and ${\mathrm{N}\mathrm{H}}_{4}^{+}$(OR = 1.97, 95% CI 1.57–2.45) than those in northern Guangdong(${\mathrm{N}\mathrm{O}}_{3}^{-}$: OR = 1.12, 95% CI 0.91–1.36; ${\mathrm{N}\mathrm{H}}_{4}^{+}$: OR = 1.24, 95% CI 1.00–1.53).

### Sensitivity analyses

The results in the sensitivity analyses were similar to those in the main analyses. When stillbirths were excluded, the association between weekly exposure to OM, ${\mathrm{S}\mathrm{O}}_{4}^{2-}$, ${\mathrm{N}\mathrm{O}}_{3}^{-}$, ${\mathrm{N}\mathrm{H}}_{4}^{+}$ and BC in early pregnancy and CHD remained stable in direction, magnitude and statistical significance (Supplementary Tables S1). The key window for exposure to PM_2.5_ components during pregnancy may still be early pregnancy, with the highest susceptibility in the first week. The estimated effect sizes for each PM_2.5_ component exposure in the first week of pregnancy were shown as follows: OM(OR = 1.94, 95% CI 1.90–1.97), ${\mathrm{S}\mathrm{O}}_{4}^{2-}$(OR = 2.29, 95% CI 2.25–2.34), ${\mathrm{N}\mathrm{O}}_{3}^{-}$(OR = 1.64, 95% CI 1.60–1.68), ${\mathrm{N}\mathrm{H}}_{4}^{+}$(OR = 1.49, 95% CI 1.45–1.53) and BC(OR = 1.97, 95% CI 1.94–2.00).

The sensitivity analysis results based on the WQS model are presented in Supplementary Fig. S1. This figure showed that PM_2.5_ components in early pregnancy were positively correlated with CHD, consistent with the results using the QGC method in the main analyses. ${\mathrm{S}\mathrm{O}}_{4}^{2-}$ may be mainly responsible for the association between PM_2.5_ and CHD, followed by BC. The corresponding contribution weights for ${\mathrm{S}\mathrm{O}}_{4}^{2-}$ and BC were 88.30% and 11.40%, respectively.

## Discussion

This is one of the few large-scale studies to investigate the association between PM_2.5_ components and CHD. Based on the data of 2,601,300 participants in Guangdong, China, we found a significant positive relationship between exposure to major PM_2.5_ constituents (OM, ${\mathrm{S}\mathrm{O}}_{4}^{2-}$, ${\mathrm{N}\mathrm{O}}_{3}^{-}$, ${\mathrm{N}\mathrm{H}}_{4}^{+}$, and BC) during early pregnancy and the risk of CHD. Among these five major PM_2.5_ components, ${\mathrm{S}\mathrm{O}}_{4}^{2-}$ emerged as the predominant contributor in the mixture effect, suggesting that it may play a central role in the positive association between PM_2.5_ exposure and CHD risk. Furthermore, a stratified analysis revealed notable regional differences in susceptibility to specific PM_2.5_ components among pregnant women across Guangdong Province.

Accumulating evidence is consistent with our results, indicating that PM_2.5_ exposure during early pregnancy is positively associated with CHD risk in offspring. A study using hospital-based registry data in Hefei found weekly air pollution exposure was positively associated with CHD^31^. Notably, a longitudinal case-control study in Guangdong, including 7055 CHD cases and 6423 controls with an average PM_2.5_ concentration of 43 μg/m^3^, found that early pregnancy may be a critical exposure window for the effect of PM_2.5_ on CHD^32^. On the other hand, a small number of studies yield opposite results. A meta-analysis found no association between prenatal PM_2.5_ exposure and CHD, and even found negative associations with certain CHD subtypes^33^. A case-control study in Beijing, China (involving 2,600 healthy infants and 360 CHD cases) found no increased CHD risk under PM_2.5_ exposure during preconception or early pregnancy, and a negative association between maternal PM_2.5_ exposure during mid-pregnancy and fetal CHD risk^34^. The reason for the different results between the above study and our study is that the previous study reported a higher level of average local PM_2.5_ concentration than our study (84 μg/m^3^ vs. 43 μg/m^3^). High levels of PM_2.5_ in Beijing could lead to spontaneous abortion and preterm birth during early pregnancy, which acted as competing risks for CHD and thus led to an observed negative association^35^. Moreover, the limited sample size in this study may hinder the detection of precise effects^36^.

The adverse effects of PM_2.5_ on pregnancy outcomes are largely determined by its chemical composition. In recent years, researchers begin to pay more attention to the associations between PM_2.5_ constituents and adverse health events, such as precocious puberty, diabetes, and macrosomia^36–38^. In this study, we employed the QGC method to estimate the joint effects of five major PM_2.5_ components on CHD, and found that ${\mathrm{S}\mathrm{O}}_{4}^{2-}$ was the principal component associated with the risk of CHD, followed by BC. This aligns with some past findings but also shows differences^16,17^. For example, a study in Chongqing, China (involving 9152 pregnant women) pointed to BC and ultrafine particles (UM), while a Chinese nationwide study (involving 4695 pregnant women) highlighted OM and BC. All three studies agree on BC’s role, but differ on the second key pollutant. This discrepancy may stem from our study’s lack of UM measurement, which could explain the absence of UM effects in our findings. Additionally, our study region has lower levels of PM_2.5_ components, with different primary constituents compared to other regions. Regional variations in PM_2.5_ composition may lead to differential pollution effects, as the health impacts of individual components can interact and produce distinct health outcomes^39^. Our findings provide novel evidence that specific PM_2.5_ component exposure during early pregnancy may increase CHD risk in offspring, with ${\mathrm{S}\mathrm{O}}_{4}^{2-}$ identified as the principal driving component. The main sources of ${\mathrm{S}\mathrm{O}}_{4}^{2-}$ are coal combustion, motor vehicle emissions, waste incineration, road dust, secondary transformation from sulfur dioxide and endogenous production in the human body^40^. BC primarily originates from combustion processes, such as industrial emissions, vehicular exhaust and residential coal burning^41^. These results suggest that pregnant women should enhance personal protective measures, including avoiding sources of combustion, and minimizing prolonged exposure to areas with heavy vehicular emissions.

The biological mechanisms linking PM_2.5_ components to CHD remain incompletely understood. Maternal exposure to ${\mathrm{S}\mathrm{O}}_{4}^{2-}$ and BC during pregnancy may put offspring at risk of CHD through pathways involving oxidative stress, inflammation, and hemodynamic alterations^42^. Studies indicated that prenatal exposure to ${\mathrm{S}\mathrm{O}}_{4}^{2-}$ may elevate the risk of CHD by affecting calcium channels in vascular smooth muscle and reducing calcium influx. This inhibits constriction of the ductus arteriosus, increases the persistence of patency-promoting factors in the fetus, and results in significant hemodynamic alterations^43,44^. ${\mathrm{S}\mathrm{O}}_{4}^{2-}$ is also associated with various circulating inflammatory markers and may play a critical role in inflammation and coagulation^45^. A cohort study revealed that maternal exposure to ${\mathrm{S}\mathrm{O}}_{4}^{2-}$ was associated with reduced activity of polymorphonuclear leukocytes. This suppresses the release of platelet-activating factors from macrophages, triggering oxidative stress and a systemic inflammatory response^46^. ${\mathrm{S}\mathrm{O}}_{4}^{2-}$ acidifies the atmospheric environment, enhancing the solubility and bioavailability of heavy metals in PM_2.5_. This may increase reactive oxygen species in the human body and thus induce oxidative stress and inflammation^47^. Studies demonstrated that maternal-inhaled carbonaceous particles may cross the placental barrier to fetal organs, and thus BC may breach the placental barrier and directly or indirectly interfere with normal fetal development^48^. Proteomic studies revealed that 13 functional proteins were significantly upregulated in the placentas of pregnant women with high BC exposure, mainly enriched in pathways related to extracellular matrix remodeling, nutrient transport, fibrin clot formation, and hemostasis regulation^49^. This elucidates the damage mechanism of BC exposure on placental structural integrity, substance exchange function, and intrauterine microenvironment homeostasis, providing pathophysiological evidence for its risk of mediating fetal cardiac developmental abnormalities. Another longitudinal study reported that exposure to BC was positively associated with elevated systemic inflammatory markers, particularly vascular endothelial growth factor, and may disrupt regulation of oxidative stress, coagulation and lipid metabolism^50^. Animal experiments suggested that nitrate may directly affect the differentiation and maturation of the embryonic cardiovascular system by interfering with estrogen receptor signaling and regulating key genes in heart development^51^. ${\mathrm{N}\mathrm{O}}_{3}^{-}$ and ${\mathrm{N}\mathrm{H}}_{4}^{+}$ in PM_2.5_ do not directly act on embryonic myocardial tissue, but interfere with placental implantation process, inhibit placental angiogenesis, and damage function of the placenta, leading to intrauterine hypoxia and disorders in the embryo^52^. This indirectly increases the risk of fetal cardiac malformations. The observed association between PM_2.5_ component exposure and CHD in this study can be explained through the above biological pathways. Future studies should enhance mechanism discussion by combining the physicochemical properties of each component with the research progress.

Our study found that CHD risk associated with exposure to PM_2.5_ components varied significantly among infants from different regions of Guangdong Province, and this variation may be influenced by air pollution sources^30^. Compared with other regions, the Pearl River Delta region is the most developed region in Guangdong, with its high degree of industrialization, dense population, traffic congestion, large resource consumption and high exposure concentration of PM_2.5_ components. The complex environmental conditions expose mothers residing in such areas to more health-harming risk factors, thereby elevating the risk of their infants developing CHD^53^. This study identified key factors underlying the association between PM_2.5_ and CHD, and assessed the susceptibility of local pregnant women to these five pollutants across different regions, thus providing valuable evidence to inform the development of targeted emission reduction policies.

This study has several limitations. First, exposure to air pollutants was assessed according to the residential district/county of pregnant women, which may not capture all sources of air pollution, such as indoor air pollution, time spent outdoors, and spatial activity patterns, potentially resulting in exposure misclassification. Second, we did not account for changes in residential district/county due to relocation during pregnancy; however, previous research indicated that only 2.6% of pregnant women moved out of their original residential area^29^. Third, due to dataset constraints, we were unable to adjust for potential confounders, such as maternal socioeconomic status, periconceptional diseases, and medication use, which may influence the observed associations.

## Conclusions

In this study, a significant association was found between exposure to PM_2.5_ components in early pregnancy and CHD. Specific components of PM_2.5_ contributed significantly to this association, with ${\mathrm{S}\mathrm{O}}_{4}^{2-}$ likely being the primary driver of the link between PM_2.5_ and CHD. In addition, the offspring in the Pearl River Delta region have a higher possibility of suffering from congenital heart disease. This study fills the gap in large-scale population-based epidemiological research on the association between PM_2.5_ components and CHD, and provides novel targets and a theoretical basis for the precise prevention and control of CHD. It is recommended to strengthen the monitoring and regulation of atmospheric ${\mathrm{S}\mathrm{O}}_{4}^{2-}$ concentration, with particular emphasis on the Pearl River Delta region. Meanwhile, targeted precise protection guidelines should be formulated for women in early pregnancy.

## Supplementary Information

Below is the link to the electronic supplementary material.

## Data Availability

Restrictions apply to the availability of these data. Data were obtained from Guangdong provincial birth registration database and are available from the authors with the permission of Guangdong Provincial Health Commission.

## Abbreviations

CHD: Congenital heart disease
PM_2.5_: Particulate matter with aerodynamic diameter < 2.5 μm
OM: Organic matter
$${\mathrm{S}\mathrm{O}}_{4}^{2-}$$: Sulfate
$${\mathrm{N}\mathrm{O}}_{3}^{-}$$: Ammonium
BC: Black carbon
OR: Odds ratio
CI: Confidence interval
IQR: Interquartile range
QGC: Quantile g-computation
WQS: Weighted quantile sum
