# Supplementary material for: The impact of PM2.5 component exposure during early pregnancy on congenital heart disease: evidence from Guangdong, China
Source: Sci Rep. 2026 Jul 28;16:27543. doi: 10.1038/s41598-026-59940-7 (PMC13538357; doi:10.1038/s41598-026-59940-7)
Supplement: Supplementary file 1 [file 41598_2026_59940_MOESM1_ESM.docx]

Appendix A. Supplementary data

**The impact of PM_2.5_ component exposure during early pregnancy on** **congenital heart disease: Evidence from Guangdong, China**

Yanbing Chen^1†^, Peiming Su^2,3†^, Qixian Gu^3^, Huili Wei^3,4^, Jialing Qiu^5*^, Guangming Tan^3**^

^1^Medical Genetic Center, Guangdong Women and Children Hospital, 521 Xingnan Road, Panyu District, Guangzhou 511442, China

^2^School of Public Health, Guangzhou Medical University, 1 Xinzao Road, Xinzao Town, Panyu District, Guangzhou 511436, China

^3^Department of Medical Information and Statistics, Guangdong Women and Children Hospital, 521 Xingnan Road, Panyu District, Guangzhou 511442, China

^4^Department of Public Health and Preventive Medicine, School of Medicine, Jinan University, Guangzhou 510632, China

^5^Department of Human Resources, Guangdong Women and Children Hospital, 521 Xingnan Road, Panyu District, Guangzhou 511442, China

* Correspondence: qiujialingcn@126.com (J. Q.), guangmingtannhh@163.com (G. T.)

† These authors contributed equally to this work.

**Contents of this file**

Table S1

Figure S1

Table S1 Logistic regression analysis of PM_2.5_ components and congenital heart disease in different pregnancy periods (excluding stillbirths)

| Gestational weeks | OM | ${SO}_{4}^{2-}$ | ${NO}_{3}^{-}$ | ${NH}_{4}^{+}$ | BC |
| --- | --- | --- | --- | --- | --- |
|  | OR [95% CI] | OR [95% CI] | OR [95% CI] | OR [95% CI] | OR [95% CI] |
| 1 | 1.94(1.90-1.97) | 2.29(2.25-2.34) | 1.64(1.60-1.68) | 1.49(1.45-1.53) | 1.97(1.94-2.00) |
| 2 | 1.91(1.88-1.95) | 2.29(2.25-2.33) | 1.61(1.57-1.65) | 1.48(1.44-1.51) | 1.97(1.93-2.00) |
| 3 | 1.88(1.84-1.91) | 2.26(2.22-2.30) | 1.57(1.54-1.61) | 1.45(1.41-1.49) | 1.95(1.92-1.98) |
| 4 | 1.82(1.79-1.86) | 2.20(2.16-2.24) | 1.52(1.48-1.55) | 1.40(1.36-1.44) | 1.90(1.87-1.94) |
| 5 | 1.77(1.74-1.80) | 2.12(2.08-2.16) | 1.47(1.44-1.51) | 1.35(1.31-1.38) | 1.85(1.82-1.89) |
| 6 | 1.76(1.73-1.80) | 2.11(2.07-2.15) | 1.48(1.44-1.51) | 1.34(1.30-1.37) | 1.84(1.81-1.87) |
| 7 | 1.74(1.71-1.77) | 2.06(2.03-2.10) | 1.46(1.42-1.49) | 1.33(1.30-1.36) | 1.80(1.78-1.83) |
| 8 | 1.74(1.71-1.77) | 2.05(2.01-2.08) | 1.45(1.41-1.48) | 1.33(1.30-1.37) | 1.82(1.79-1.85) |
| 9 | 1.72(1.69-1.75) | 2.03(1.99-2.06) | 1.40(1.36-1.43) | 1.28(1.25-1.31) | 1.81(1.78-1.84) |
| 10 | 1.68(1.65-1.71) | 2.00(1.96-2.03) | 1.34(1.31-1.37) | 1.23(1.19-1.26) | 1.78(1.75-1.81) |
| 11 | 1.71(1.69-1.75) | 2.07(2.03-2.11) | 1.38(1.35-1.41) | 1.27(1.24-1.30) | 1.83(1.80-1.86) |
| 12 | 1.76(1.72-1.79) | 2.14(2.10-2.18) | 1.38(1.35-1.41) | 1.27(1.24-1.30) | 1.88(1.85-1.91) |

*OM: organic matter; ${SO}_{4}^{2-}$: sulfate;${NO}_{3}^{-}$: nitrate; ${NH}_{4}^{+}$: ammonium; BC: black carbon. All the above models were adjusted according to the age of the mother, gravidity, parity, neonatal gender, gestational age, conception season and temperature

Fig. S1 The contribution weights of the five main components of PM_2.5_ to the overall effect on congenital heart disease.
